# 2214 Nutritional outcomes in pediatric oncology patients less than 3 years of age

**DOI:** 10.1017/cts.2018.293

**Published:** 2018-11-21

**Authors:** Daniel Runco, Ann Mertens, Karen Wasilewski-Masker

**Affiliations:** Department of Pediatrics, Children’s Healthcare of Atlanta, Emory University

## Abstract

OBJECTIVES/SPECIFIC AIMS: Brain tumors are the most common solid tumor diagnosed in children and their location predisposes patients to oromotor dysfunction leading to feeding difficulties. Brain tumor patients experience feeding difficulties more frequently than other pediatric malignancies, largely due to central nervous system directed chemotherapy, radiation, and surgery. Treatment increases the risk of malnutrition and increases risk for infection, ICU admissions, and death. Infants and children (less than 3 years of age) are at higher risk for malnutrition due to rapidly changing nutritional requirements and the underdevelopment of motor skills. Incidence and prevalence of malnutrition in pediatric cancer patients is not well known. METHODS/STUDY POPULATION: This is an observational, retrospective study of our center’s pediatric cancer patients. Patients are classified by diagnosis, treatment intensity (ITR-2), vital status, and heights and weights (with standardized *Z*-scores) will be recorded with through 2 years after diagnosis. Adaptation of Intensity of Treatment Rating ITR-2. Nutrition consultation, ICU admissions, and use of parenteral or enteral nutrition will be recorded. Weight loss greater than a 5-percentile point change or *Z*-score decrease greater than 0.5 will be treated as a binary outcome and considered significant weight loss. RESULTS/ANTICIPATED RESULTS: Preliminary analysis has identified 465 eligible subjects as described above: brain tumor (n=45) and nonbrain tumor patients (n=420). Patient Schema. This study is still in progress and aims to better identify incidence of malnutrition during pediatric cancer therapy. It is expected that a greater number of nonbrain tumor patients compared with brain tumor patients will be malnourished as defined by decrease in *Z*-score greater than 0.5 at any point in therapy or falling below the 5th percentile of weight for age. Weight loss will be associated with higher number of ICU admissions and higher treatment intensity score. Finally, we expect to find that patients with a larger decrease in Z-score for age will be more likely to die during therapy. DISCUSSION/SIGNIFICANCE OF IMPACT: In addition to this study being valuable in better defining incidence of malnutrition, this data will serve as preliminary data in defining target populations for focused nutritional intervention during cancer therapy. Using this established and published intensity rating scale, we may be able to identify better methods of identifying and preventing malnutrition during cancer therapy.

